# Breast mucosa-associated lymphoid tissue lymphoma: A case report and literature review

**DOI:** 10.1097/MD.0000000000037895

**Published:** 2024-04-19

**Authors:** Siyi Luo, Xinyue Zhang, Zhichun Wang

**Affiliations:** a Department of Breast, Jiujiang NO.1 People’s Hospital, Jiujiang, People’s Republic of China; b Department of Pathology, Jiujiang NO.1 People’s Hospital, Jiujiang, People’s Republic of China.

**Keywords:** breast, case report, lymphoma, mucosa-associated lymphoid tissue

## Abstract

**Background:**

Mucosa-associated lymphoid tissue (MALT) lymphoma, also known as extranodal marginal zone lymphoma, is more commonly detected in the stomach and rarely in the breast. Our study presented a clinical and pathological examination of a patient diagnosed with breast MALT lymphoma, supplemented with pertinent research, to offer guidance for the diagnosis and treatment of this condition.

**People concerns:**

The occurrence of breast MALT lymphoma has risen in the past decade, but its etiology, progression and treatment response are less well-studied.

**Diagnosis:**

Breast MALT lymphoma was diagnosed by excisional biopsy and histopathology.

**Interventions:**

Following breast MALT lymphoma diagnosis, the patient was transferred to the hematology department for further treatment, and she made the decision to continue observing.

**Outcomes:**

After 3 months of observation, the patient remained asymptomatic.

**Conclusion:**

Breast MALT lymphoma is an indolent disease with an asymptomatic presentation, There are no standardized treatment guidelines for breast MALT lymphoma, treatment must be tailored to the patient willingness to treat and the severity of the disease. Hence, in order to give patients a better chance of cure, more research is needed to explore its pathogenesis and more clinical trials are needed investigate the treatment of this disease.

## 1. Introduction

Breast lymphoma (BL) is an uncommon malignant tumor that occurs in the breast. The most prevalent type of BL is diffuse large B-cell lymphoma.^[1]^ Additionally, breast mucosa-associated B-cell lymphoma is a less common form of BL, making up approximately 9% of all BLs.^[2]^ In this paper, we report a case of breast mucosa-associated lymphoid tissue (MALT) lymphoma and the pathogenesis, diagnosis and treatment of breast MALT lymphoma are discussed by combining with previous relevant literature.

## 2. Case presentation

**Figure 1.**
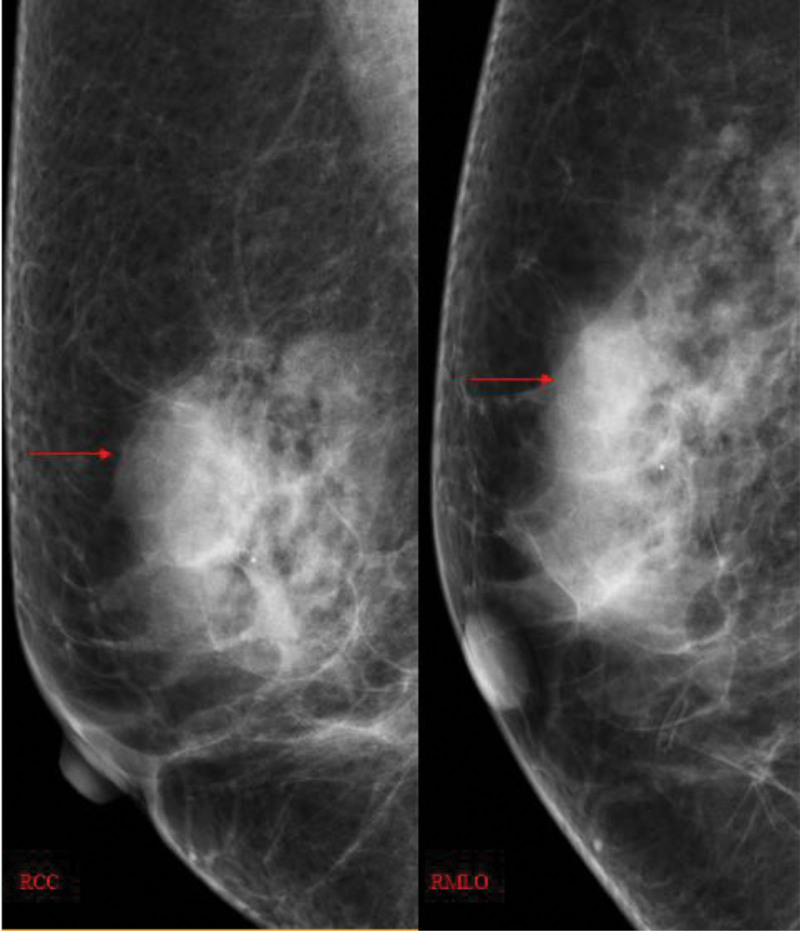
A 1.6-cm nodule at the edge of the gland superior to the lateral side of the right breast.

**Figure 2.**
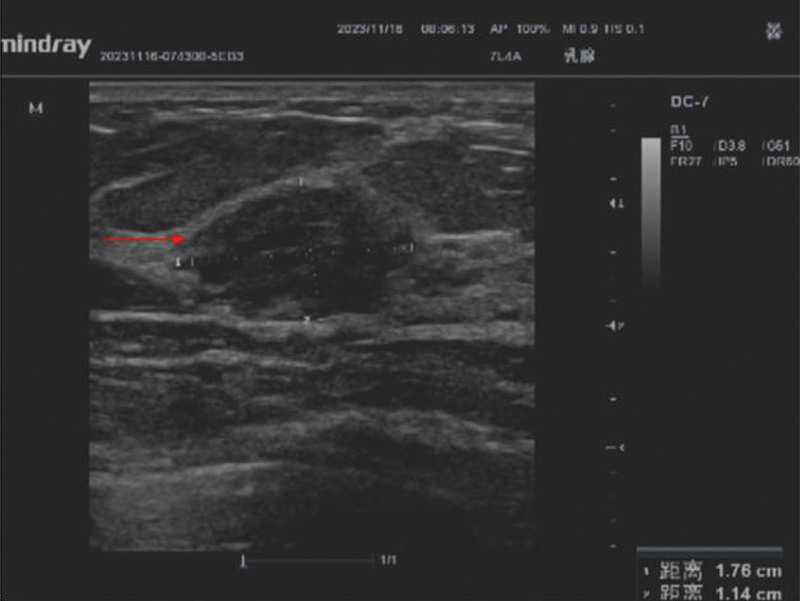
A hypoechoic mass measuring 17.6 mm in the upper outer quadrant of the right breast.

**Figure 3.**
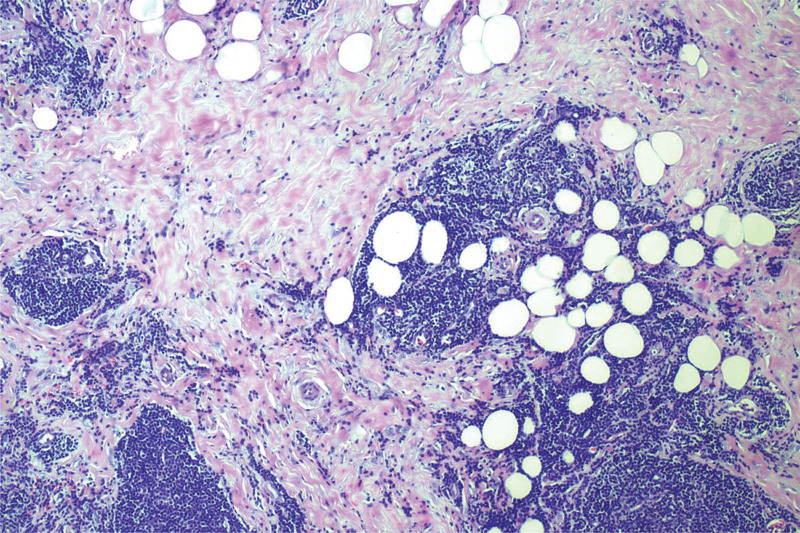
The HE stain show the infiltration of the adipose tissue in the breast by lymphocytes of MALT lymphoma. MALT = mucosa-associated lymphoid tissue.

**Figure 4.**
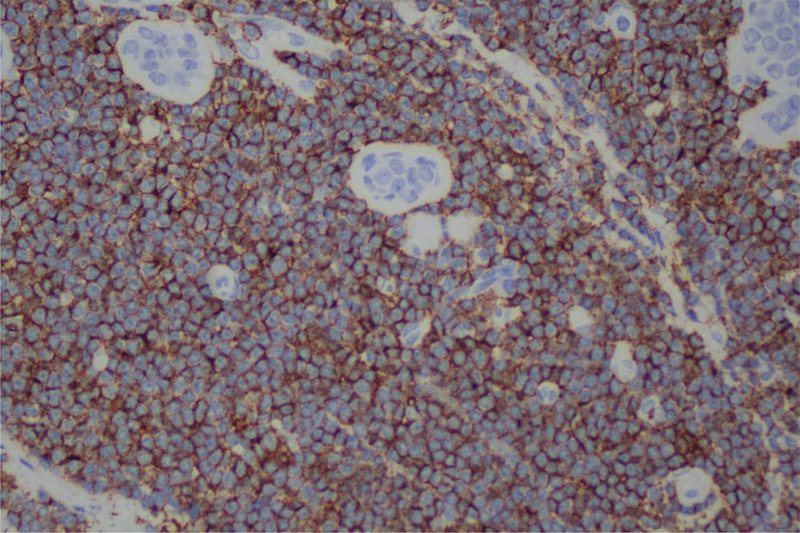
The results of immunohistochemical staining showed that the tumor expressed CD20.

A 64-year-old asymptomatic Chinese female patient underwent a physical examination at our hospital. The color doppler ultrasound indicated the presence of a hypoechoic mass measuring 17.6mm in the upper outer quadrant of the right breast. The boundaries of the mass were unclear, and the shape was irregular. Its aspect ratio was <1, and there was no noticeable blood flow signal (Fig. 1). A routine mammogram revealed a 1.6-cm nodule at the edge of the gland superior to the lateral side of the right breast (Fig. 2). After admission, blood cell analysis: the white blood cell count was 35.12*10^9/L, the lymphocyte percentage was 75%, and the absolute lymphocyte count was 26.35*10^9/L. The possibility of breast fibroadenoma was preliminarily considered, then we performed a lumpectomy. The lesion was sent to the pathology department. Microscopically, we can see mononuclear B cells, small lymphocytes and scattered immune blast cells. Some of the tumor cells infiltrated the surrounding adipose tissue (Fig. 3).And the immunohistochemical results showed that Mum-1(−),BCL-2(+),CyclinD1(−),TdT(−),PAX-5(+),CD21(−),CD23(−),CD5(-),CK(-),CD20(+),CD43(Focal+),CD3(−),EBER(−),ALK(−),CD30(−),C-myc(Focal+),CD10(−),Bcl-6(−),CD20(+) (Fig. 4). After surgery, the patient underwent PET-CT: Lymph node metabolism was slightly higher in multiple groups of the whole body [bilateral neck (I, II, III, IVa, V), right armpit, right outer upper breast quadrant], which was consistent with lymphoma-involved lesions, and the Deauville scale was 2 to 4. The patient was then transferred to the hematology department for subsequent specialized treatment. Considering the patient treatment intention, she decided to proceed with observation, and after 3 month of follow-up monitoring, the patient remained asymptomatic.

## 3. Discussion

MALT lymphoma stands out as a distinctive form of lymphoma, setting it apart from other slow-growing B-cell lymphomas. Its initial identification can be attributed to the work of pathologists Peter Isaacson and Dennis Wright, who made the discovery while studying lymphomas affecting specific gastric tissues.^[3]^ Based on continuous understanding and exploration, MALT lymphoma is one of the most common lymphomas, accounting for 7% to 8% of lymphomas, and the number of female patients is more than that of male patients, and the median age of diagnosis is 65 years.^[4,5]^ The age of the patient in our case is consistent with this.^[6]^ In addition to the stomach, MALT lymphoma can be found in the lungs, small intestine, salivary glands, and thyroid gland.^[7]^ The occurrence of MALT lymphoma in the breast is also relatively rare.

MALT tumor cells express B-cell-associated antigens, such as CD20 and CD79a, which are positive in MALT lymphomas. And when we exclude other types of lymphoma, the negative results of other lymphoma-specific antibody markers are very important. For example, CD5 (−) can rule out small lymphocytic lymphoma, Cyclin D1 (−) can rule out mantle cell lymphoma.

Autoimmune diseases, like Sjogren syndrome and Hashimoto thyroiditis, frequently coexist with MALT lymphoma during its development.^[5]^ The occurrence of gastric MALT lymphoma is closely linked to the chronic inflammation caused by *helicobacter pylori (H pylori*). Several studies state that *H pylori* plays a crucial role in the development of gastric MALT lymphoma, and multiple guidelines now emphasize that curing *H pylori* is the established primary approach for patients with Hp-positive gastric MALT lymphoma.^[8,9]^Recent studies have reported that MALT lymphoma, which is caused by *H pylori*, is characterized by the infiltration of immune effector cells and an increase in inflammation-promoting mediators. As a result, various stages of the disease are initiated. It has been observed that the CD40/40L signaling pathway, dependent on T cells, and engagement of the B-cell receptor play a crucial role in this model. Additionally, the activation of NF-κB is essential for facilitating this process.^[10–13]^

Breast MALT lymphoma is a painless breast mass that is typically discovered incidentally. Many patients do not experience noticeable symptoms, as described in this article. The exact cause of MALT lymphoma is still uncertain, although some researchers suggest that high-risk factors for the disease may include hepatitis C or chronic inflammation related to autoimmune disorders.^[14]^ Another theory suggests a relationship between the occurrence of MALT lymphoma and the regulation of immune function by sex hormones.^[15,16]^ One of the prominent genetic features of MALT lymphoma is the frequent occurrence of t(11;18)(q21;q21) translocation, particularly in gastric MALT lymphoma. This chromosomal abnormality is detected in 25% to 48% of cases of MALT lymphoma with involvement of the stomach.^[17]^ In addition to these findings, reported karyotype alterations include t(1;14)(p22;q32),t(14;18)(q32;q21),t(3;14)(q27;q32), and t(3;14)(p14.1;q32).^[12,13]^ However, Bcl-1,bcl-2,bcl-3,and bcl-6 rearrangements were not found to be associated with MALT lymphoma.^[18]^ Furthermore, MALT lymphoma has been observed to transform into diffuse large B-cell lymphoma.^[19]^

Breast MALT lymphoma is a rather uncommon phenomenon in breast tumor. Table 1 summarizes characteristics of breast MALT lymphoma cases available in the English language from the past 2 decades. We determined that the presence of SS is associated with the development of breast MALT lymphoma. Retamozo et al found that individuals with primary Sjogren syndrome have a significantly increased risk of lymphoma, ranging from 10 to 40 times higher than that of healthy individuals. This risk surpasses that of systemic lupus erythematosus and rheumatoid arthritis.^[40]^ Nevertheless, the exact correlation between breast MALT lymphoma and SS remains uncertain. So it is imperative to provide female SS patients with more diligent monitoring of their breasts to ensure early detection of BL development.

**Table 1:** Reports on breast MALT lymphoma in the past 20 yr.

Study	Age	Medical history	Location	Treatment	OS	PFS
Batstone et al, 2003^[20]^	87	NA	Left breast, axillary node, Eye, left submandibular, left submental and left parotid areas	RT	>11 mo	NA
Kambouchner et al, 2003^[21]^	37	SS	Left breast, axillary node microcalcifications	Lumpectomy	>42 mo	>42 mo
Kuper-Hommel et al, 2003^[22]^	65	Myocardial infarction and acute congestive heart failure	Right breast	Mastectomy with axillary lymph node dissection + RT + CHOP*3	13 mo	NA
Raderer et al, 2005^[23]^	72	NA	Both breasts, multiple subcutis lesions	Oxaliplatin*4	NA	12 mo
Taeda et al, 2006^[24]^	84	NA	Right breast, axillary node	Mastectomy and axillary dissection + rituximab	>18 mo	NA
Welsh et al, 2006^[25]^	66	NA	Right breast	Lumpectomy + RT	>3 yr	>3 yr
Anavekar et al, 2008^[26]^	56	Breast cancer	Left breast	Breast-conserving surgery and a sentinel lymph node biopsy + RT + Tamoxifen	>2 yr	>2 yr
Rajendran et al, 2008^[27]^	66	NA	Left breast	RT	>7 yr	>7 yr
Julen et al, 2009^[28]^	86	NA	Right breast	Mastectomy and axillary dissection	5 yr	5 yr
Ghetu et al, 2011^[29]^	77	NA	Left breast	Lumpectomy	NA	11 mo
Arslan et al, 2012^[30]^	69	NA	Left breast	Lumpectomy + CHOP*6 + rituximab *2	NA	NA
Nassif et al, 2013^[31]^	18	Idiopathic cardiomyopathy with heart transplant	Right breast	Tacrolimus and azathioprine	>6 mo	>6 mo
Kim et al, 2015^[32]^	55	NA	Both breasts	Total mastectomy of both breasts	>9 mo	>9 mo
Hissourouiii et al, 2016^[33]^	40	NA	Left breast	Lumpectomy + RT	>3 mo	>3 mo
Amalia et al, 2017^[34]^	67	SS	Both breasts	NA	NA	NA
Belfeki et al, 2019^[35]^	65	SS	Right breast	NA	NA	NA
Ludmir et al, 2019^[36]^	NA	NA	Both breasts	Observation	NA	67.2 mo
Ludmir et al, 2019^[36]^	NA	NA	Both breasts	Observation	NA	22.6 mo
Ludmir et al, 2019^[36]^	NA	NA	Right breast	Single-agent rituximab	NA	1.6 mo
Ludmir et al, 2019^[36]^	NA	NA	Left breast	RT	NA	47.8 mo
Ludmir et al, 2019^[36]^	NA	NA	Right breast	RT	NA	43.6 mo
Ludmir et al, 2019^[36]^	NA	NA	Right breast	RT	NA	82.6 mo
Ludmir et al, 2019^[36]^	NA	NA	Left breast	RT	NA	34.3 mo
Ludmir et al, 2019^[36]^	NA	NA	Left breast	RT	NA	73.5 mo
Ludmir et al, 2019^[36]^	NA	NA	Left breast	RT	NA	74.0 mo
Ludmir et al, 2019^[36]^	NA	NA	Right breast	RT	NA	7.3 mo
Ludmir et al, 2019^[36]^	NA	NA	Left breast	Lumpectomy + RT	NA	18.8 mo
Ingravallo et al, 2020^[37]^	57	SS	Right breast	NA	NA	NA
Anendaga et al, 2022^[38]^	63	NA	Right breast	Systemic chemotherapy with rituximab*4	>2 yr	>2 yr
Hussain Dalal et al, 2023^[39]^	70	SS and Stage 1 renal cell carcinoma	Left breast	Lumpectomy	NA	NA

CHOP = cyclophosphamide, doxorubicin, vincristine and prednisone, MALT = mucosa-associated lymphoid tissue, NA = not available, OS = overall survival, PFS = progression-free survival, RT = radiation therapy, SS = Sjogren syndrome.

The treatment and management of breast MALT lymphoma is currently unclear. However, there have been no extensive studies examining the standard treatment regimen. Therefore, it may be beneficial to consider treatment strategies for MALT lymphoma in other areas of the body while treating breast MALT lymphoma.

The approach to treating MALT lymphomas differs based on the location of the tumor. In the case of gastric MALT lymphoma, the recommended treatment options include curing *H pylori* infection and using systemic chemotherapy or radiotherapy.^[41,42]^ Local treatment, such as radiotherapy, is typically the primary approach for early non-gastric MALT lymphoma, with a 5-year OS(overall survival) reaching up to 96.6%.^[43]^ The side effects in general were minimal. If the disease worsens following radiotherapy or if the patient is originally diagnosed with advanced lymphoma, systemic therapy becomes the primary treatment approach.

There is currently no agreement on the recommended protocol for treating recurring or advanced MALT lymphoma. Rituximab, a monoclonal CD20 antibody, is a frequently employed therapy either on its own or alongside chemotherapy to cure lymphoma.^[44]^ In their study, Professor Zucca demonstrated that the combination therapy of rituximab increased progression-free survival without a significant impact on OS.^[45]^ Therefore, for patients who are expected to live longer and can tolerate the treatment well, the combination of rituximab may be taken into consideration in order to enhance long-term survival outcomes. The CHOP (Cyclophosphamide, Doxorubicin, Vincristine and Prednisone) regimen is the established initial chemotherapy for patients with advanced lymphoma. Professor Rummel phase III clinical trial has proven that the bendamustine and rituximab (BR) is just as effective as the rituximab plus CHOP (R-CHOP) combination in treating advanced lymphoma patients. Furthermore, BR has shown an improved progression-free survival rate and fewer adverse effects.^[46]^ Professor Flinn BRIGHT study demonstrated that not only BR not inferior to R-CHOP, but it was also not inferior to rituximab plus cyclophosphamide, vincristine and prednisone (R-CVP) in treating advanced lymphoma.^[47]^ Furthermore, certain clinical trials have demonstrated positive outcomes with lenalidomide, ibrutinib, autologous stem cell transplantation, and other therapies. These modalities can also serve as viable alternative treatment choices for eligible patients.

This case study involves a patient with breast MALT lymphoma who underwent local resection and was closely monitored for 3 months, exhibiting a positive prognosis with no relapse or metastasis. The chosen treatment strategy may have been suboptimal due to the patient reluctance. According to established guidelines and previous research, radiotherapy and chemotherapy are commonly advised for treating breast MALT lymphoma. Typically, radiotherapy as a local intervention leads to favorable results in early-stage breast MALT lymphoma, while systemic interventions such as chemotherapy play a crucial role in advanced cases. MALT lymphoma does not generally require surgical intervention. Nevertheless, small biopsy samples and fine needle aspirations might not yield enough tissue for an accurate diagnosis. In such cases, a local surgical biopsy with less invasiveness could be a viable option to acquire more biopsy tissue.

## 4. Limitations of the study

There are some limitations to this study. Firstly, it had a small sample size and a short follow-up duration. The only treatment provided was surgical resection, without any specific interventions. Despite a review of relevant literature, there is limited high-quality evidence available for treating this condition. It is imperative to conduct large-scale, multicenter clinical trials in order to develop evidence-based medical protocols. These initiatives will help in standardizing the diagnosis and treatment of breast MALT lymphoma, ultimately improving patient outcomes.

## 5. Conclusions

Breast MALT lymphoma is a rare form of breast malignant tumor. Its clinical symptoms often present as a painless mass in the breast, which can be mistaken for breast cancer. To properly diagnose this condition, immunohistochemistry of tumor tissue is necessary. Currently, researches have primarily focused on the pathogenesis and treatment of gastric MALT lymphoma, while there is a lack of standardized treatment and follow-up protocols for breast MALT lymphoma. Additionally, it is unclear whether estrogen plays a role in its development and if there are specific treatments targeting this aspect. In recent years, the incidence of BL has been on the rise. Therefore, it is important to remain vigilant when diagnosing and treating BL, continuously investigate its pathogenesis, and develop appropriate diagnostic methods and treatment strategies that are tailored to each patient needs in order to maximize their chances of recovery.

## Author contributions

**Data curation:** Siyi Luo.

**Formal analysis:** Siyi Luo.

**Investigation:** Siyi Luo, Xinyue Zhang.

**Resources:** Siyi Luo, Xinyue Zhang.

**Supervision:** Zhichun Wang.

**Writing – original draft:** Siyi Luo.

**Writing – review & editing:** Siyi Luo, Zhichun Wang, Xinyue Zhang.
